# Recent advances on the methods developed for the identification and detection of emerging contaminant microplastics: a review

**DOI:** 10.1039/d3ra05420a

**Published:** 2023-12-12

**Authors:** Preethika Murugan, Pitchiah Sivaperumal, Surendar Balu, Sandeep Arya, Raji Atchudan, Ashok K. Sundramoorthy

**Affiliations:** a Institute of Materials Resource Management, Universität Augsburg Am Technologiezentrum 8 86159 Augsburg Germany; b Marine Biomedical Research Lab & Environmental Toxicology Unit Cellular and Molecular Research Centre, Saveetha Dental College and Hospitals, Saveetha Institute of Medical and Technical Sciences, Saveetha University Chennai 600077 Tamil Nadu India; c Department of Physics, University of Jammu Jammu Jammu and Kashmir 180006 India; d School of Chemical Engineering, Yeungnam University Gyeongsan 38541 Republic of Korea; e Centre for Nano-Biosensors, Department of Prosthodontics, Saveetha Dental College and Hospitals, Saveetha Institute of Medical and Technical Sciences, Saveetha University Chennai 600077 Tamil Nadu India ashok.sundramoorthy@gmail.com

## Abstract

The widespread use of plastics, popular for their versatility and cost-efficiency in mass production, has led to their essential role in modern society. Their remarkable attributes, such as flexibility, mechanical strength, lightweight, and affordability, have further strengthened their importance. However, the emergence of microplastics (MPs), minute plastic particles, has raised environmental concerns. Over the last decade, numerous studies have uncovered MPs of varying sizes in diverse environments. They primarily originate from textile fibres and cosmetic products, with large plastic items undergoing degradation and contributing as secondary sources. The bioaccumulation of MPs, with potential ingestion by humans through the food chain, underscores their significance as environmental contaminants. Therefore, continuous monitoring of environmental and food samples is imperative. A range of spectroscopic techniques, including vibrational spectroscopy, Raman spectroscopy, Fourier-transform infrared (FT-IR) spectroscopy, hyperspectral imaging, and nuclear magnetic resonance (NMR) spectroscopy, facilitates the detection of MPs. This review offers a comprehensive overview of the analytical methods employed for sample collection, characterization, and analysis of MPs. It also emphasizes the crucial criteria for selecting practical and standardized techniques for the detection of MPs. Despite advancements, challenges persist in this field, and this review suggests potential strategies to address these limitations. The development of effective protocols for the accurate identification and quantification of MPs in real-world samples is of paramount importance. This review further highlights the accumulation of microplastics in various edible species, such as crabs, pelagic fish, finfish, shellfish, American oysters, and mussels, shedding light on the extreme implications of MPs on our food chain.

## Abstract 2

This review highlights the range of spectroscopic techniques, methods and tools developed for microplastics separation, analysis and their accumulation in various edible species and implications on our food chain.

## Graphical abstract

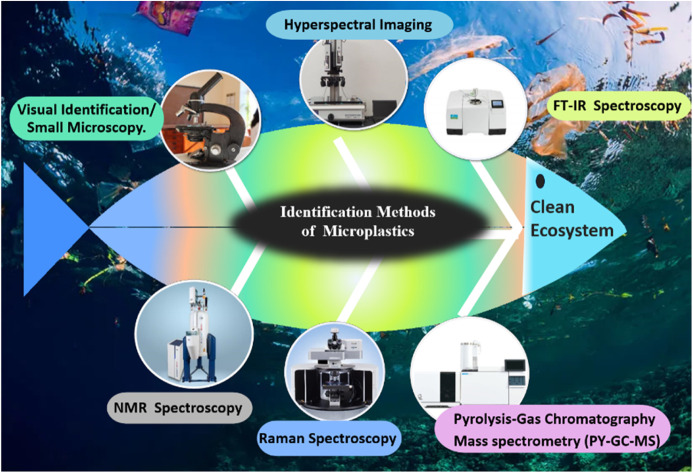

## 1. Introduction

In recent years, our ecosystem has faced an array of pressing environmental threats, most notably the looming specter of climate change,^1^ driven in part by the pervasive presence of pollutants. Amid this spectrum of pollutants, plastics have emerged as an alarming and growing concern for the environment. Plastics have integrated themselves into every facet of our daily lives, from the automobile industry to packaging, electronic appliances,^2^ construction,^3^ furniture,^4^ safety guards, sports equipment,^5^ and beyond. Astonishingly, the global production of plastics has reached a staggering 300 million tons per year, with nearly 13 million tons finding their way into our water bodies.^6^ This contribution of plastics is predicted to increase, in the form of plastic debris, to 250 million by the year of 2025.^7^ The production of plastics has grown rapidly owing to the development of large-scale industries since 1950. The manufacturing and consumption of plastics have increased from 1.7 to 335 million tons from 1950 to 2016.^8^ As a result, 8 million tons of plastics are being discarded annually. Among that, 1% of the total comprises small plastic fragments. If left unaddressed, this trend will result in a doubling of plastic pollution by 2030.^9,10^ Furthermore, owing to the disintegration of big particles, the formation of immeasurable plastic waste in marine environments may continue for decades. Such microplastics (MPs) are consumed by aquatic organisms or animals, which can be life-threatening.^10^

MPs are pieces of plastic smaller than five millimetres in size, and they may have a devastating effect on ecosystems. They pose risks to animals, alter food webs, and taint natural areas. Since they stick around for a while, they end up posing a serious ecological risk. These pollutants must be identified and quantified in the samples of interest. In the past 50 years, increasing attention has been focused on large plastic debris. The tiny fragments formed by the degradation of larger plastic waste materials affect the sea and soil quality.^11^ However, it has not received much attention by the environmental activists and researchers. Mostly, these synthetic plastic particles will not undergo biodegradation, and it can be only broken down by mechanical action. Therefore, these MPs significantly contributed to the environmental contamination.^12,13^

**Fig. 1:**
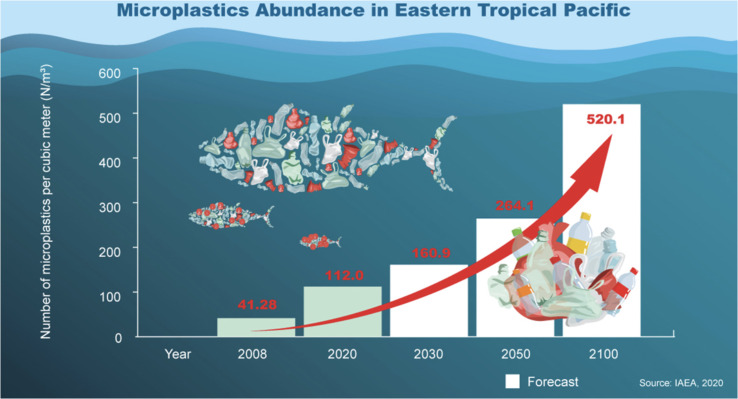
Microplastic statistics in the Eastern Tropical Pacific. Reproduced from ref. 136 with permission from International Atomic Energy Agency, copyright 2023.

In recognition of the gravity of this issue, the United Nations took a historic step in 2017, adopting a resolution on Marine Litter and MPs, with signatures from nearly 200 countries. These MPs are very small particles (size less than 5 mm), while plastic particles that are less than 1 mm are called nanoplastics.^14,15^ The plastic products could not be degraded by weathering and ageing, leading to their accumulation and persistence in the aquatic region.^16^ Moreover, the prevalent MPs were ingested by most of the aquatic species. In our review, we explore the identification and quantification of contaminants in samples of interest, shedding light on the far-reaching consequences of plastic pollution on our environment and its inhabitants. Our investigation centres on the analysis of research papers published from 2019 to 2023, with a specific focus on comprehending the scope and effects of MPs pollution that are a matter of urgent concern. We had used Web of Science and Scopus databases with the Keywords of “microplastics”. Throughout this study, we present novel insights and emphasize the research significance that will contribute to the ongoing global efforts to combat this environmental challenge (Fig. 1).

## 2. Classification of MPs

MPs are obtained from various sources and can be classified into two categories, such as primary and secondary MPs.^17^ Primary MPs are used as an additive in hygienic products, cosmetics, and industrial products (*e.g.*, paints).^18^ Secondary MPs are obtained due to the degradation of bulk plastics by weathering, mechanical crushing, and aging. Apart from this, MPs are also generated from other sources and pathways, such as natural calamities like wildfire, storms, broken parts of ship and fishing accessories.^19^

In developing countries, the waste management system is poor and most of the plastic wastes are dumped into empty landfills. The dumped plastics are exposed to sunlight, microorganisms, air and mechanical stress, which cause the bulk plastics to break down into MPs.^20^ Examination of the organic-rich materials, such as compost and soil sediment, is still in its initial stages. Thus, standardized methods for the identification and quantification of MPs are still under developmental stage. Somehow, the soils act as a sink for MPs for extended periods of time.^21^ The highly dense polymers will remain inside the soil for several years, and penetrate the soil layers deeply. The MPs (which are lighter in weight) can be taken away by wind and water to other terrestrial regions or to water bodies.^22^ Finally, MPs may reach the human system by contaminated air inhalations with MPs.^23^ The utility life of plastics may differ from one to fifty years, which depended upon the usage time before they are discarded. In the context of plastic waste management, the following statistics have been determined: a mere 9% of plastic materials were subjected to recycling efforts, while 12% were utilized for energy recovery purposes. A further 8% are designated for landfill disposal, leaving a substantial 71% of plastics dispersed into the environment.^24,25^ Several experimental trials had been carried out to evaluate the effects of plastics on the environment, especially through biota. Due to its small size, MPs can be indirectly ingested by marine organisms, which causes physical harm and toxic effects.^26^

**Fig. 2:**
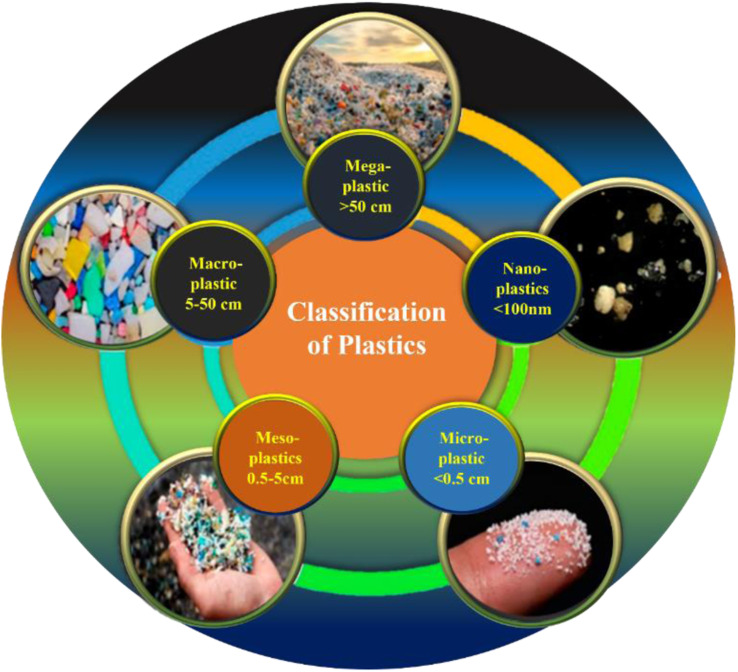
Classification of various plastics found in the environment.

A crucial point in the detection of MPs is the collection of environmental samples, pre-treatment procedures, and positive identification in the marine and soil sediments. These steps had created more complications on the effective identification process. The size of the plastics (Fig. 2) played an important role in the ingestion by various organisms. Phuong *et al.*^27^ reported that the ingestion of MPs is more dependent on the particle size than the other factors. For instance, Van Cauwenberghe *et al.*^28^ tested the MPs ingestion on lugworms where it had consumed MPs, rather than macro plastics. In addition, Gray and Weinstein reported that the mortality rate of dagger blade glass shrimps was high when they consumed MPs fibres, rather than spheres. These studies indicated the importance of the size and morphologies of MPs that could be potentially harmful to the organisms.^29^ An extensive source of information about the MPs is available, which includes its occurrence, difficulties in the evaluation, tracking of the MPs in the surroundings, non-uniformity in the field studies, *etc.*^30^ Therefore, there is a need to establish a standard protocol for the examination, assessment and characterization of MPs, which must include visualization and treatment methods. The methods developed for the MPs investigation are at its infant stage.^31^ The challenging factors of plastics is that they consist of different polymeric materials with various sizes, shapes, different composition and additives. It is required to develop new identification methods for MPs, which should be simple, rapid, and low cost. The most identified MPs in the environment are polyethylene (PE), high density polyethylene (HDPE), low density polyethylene (LDPE), polypropylene (PP), polystyrene (PS), polyamide (PA) and polyethylene terephthalate (PET).^32^

Herein, we have summarized and discussed the various techniques used to report on the examination, sample treatment, spot out and assessment of MPs contamination levels in various environmental samples (water, air, soil and biota). Specifically, identification of MPs by Raman spectroscopy, Fourier transform infrared (FT-IR) spectroscopy, hyper spectral imaging, Nuclear magnetic resonance (NMR) and pyrolysis-gas chromatography-mass spectrometry (PY-GC-MS) was highlighted. This review also discussed the overall status of identification methods developed for MPs, along with their advantages and disadvantages (Table 1). In addition, the essential factors that should be considered in the development of new methods for the identification and quantification of the MPs were discussed with possible ways to overcome such complications.

**Table 1:** General analytical methods reported for the detection of MPs and their advantages and disadvantages

S. No.	Methods and techniques	Sample preparation	Advantages	Disadvantages	References
1	Microscopy supported	(i) Prepare a suitable sample	(i) Capable for pre-sorting of samples for corresponding analysis	(i) Misconception of tiny and translucent particles	129
		(ii) Apply conductive coating if needed	(ii) Common people can easily sort out	(ii) No chemical characteristic can be identified	
		(iii) Optimize sample thickness and sectioning	(iii) Inexpensive		
		(iv) Use proper imaging and analytical settings			
2	FTIR spectroscopy	(i) For dry and solid samples, use KBr pellets	(i) Competent identification in environmental matrices	(i) Organic interference may lead to misconception	130
		(ii) Avoid contamination and air exposure	(ii) High robustness	(ii) Good results are obtained for dry samples	
		(iii) Control sample thickness	(iii) Sensitivity is less towards auto fluorescence than Raman		
		(iv) Record a clean background spectrum			
3	Raman spectroscopy	(i) Clean sample surface	(i) Well dimensional resolution than FT-IR.	(i) Sample detection is affected by auto fluorescence	131
		(ii) Use stable substrates	(ii) Size less than 1 μm can be measured	(ii) Time delayed	
		(iii) Optimize laser power and wavelength	(ii) Good sensitivity		
		(iv) Choose appropriate excitation source			
4	Hyper spectral imaging	(i) Stabilize the sample	(i) The dimensional property obtained elaborately	(i) High cost	132
		(ii) Capture a spectral image sequence	(ii) Segmentation is accurate and images are well-defined	(ii) Complication in operating	
		(iii) Apply calibration standards			
		(iv) Process and analyse data for insights			
5	NMR spectroscopy	(i) Use pure samples	(i) Exact quantification of particles can be identified	(i) Low sensitivity	87
		(ii) Dissolve in NMR solvent		(ii) Expensive instrument	
		(iii) Adjust concentration (1–10 mg mL^−1^)			
		(iv) Use clean NMR tubes			
6	Pyrolysis gas chromatography	(i) Analyze solid or polymer samples	(i) Polymer type can be identified along with the additives added to it	(i) Time delayed	133
		(ii) Use a small sample amount		(ii) Prior particle selection is done before analysis	
		(iii) Set pyrolysis conditions			
		(iv) Connect to a gas chromatography			

## 3. Properties of MPs

There are several kinds of MPs with densities ranging from (0.9–2.2 g cm^−3^), and they are distinguished by mass (light or heavy) and physical flexibility (hard or soft).^33^ Depending on the sources of the plastics, the appearance of MPs would vary based on the colour, such as red, blue, green, black and others, which can be visualized by microscope. Next, different shapes of MPs have been reported, including two-dimensional (*e.g.*, rectangle, circle, polygonal) and three-dimensional structures (*e.g.*, spherical, pellet, pyramidal), and many other irregular shapes. Wang *et al.*^34^ already discussed the behaviour of MPs in water. However, the size factor of the MPs was not taken into account, where it played an important role in the stability of both micro- and nanoplastics in the footings of agglomeration and dispersion. According to Mintenig *et al.*,^35^ the most prevalent MPs in water are PE and PP, which are hydrophobic materials and have a tendency to agglomerate in water. However, the micro- and nanoplastics had substantial effects on the stability of these agglomerations in water.

## 4. Fragmentation of plastics

**Fig. 3:**
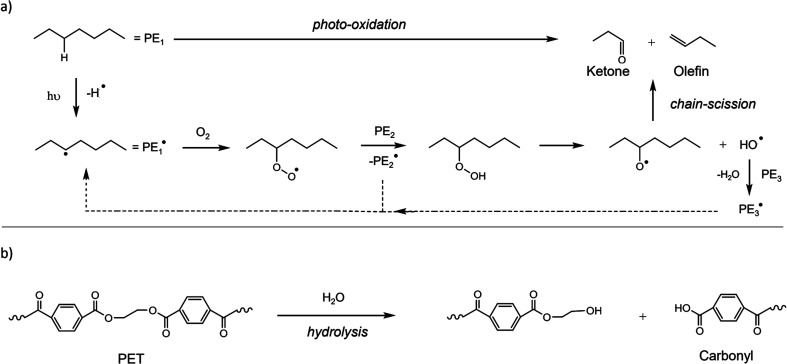
General mechanisms for the degradation of polymers. (a) Photo-degradation of PE by UV irradiation and (b) degradation of PET by hydrolysis. Reproduced from ref. 137 and 138 with permissions from Elsevier, copyright 2019; and the Royal Society of Chemistry, copyright 2015.

The fragmentation pattern of macro-sized plastics takes place by several routes (independently or jointly) such as photo-oxidation *via* Ultra-Violet (UV) light, hydrolysis, mechanical action due to scrape or water agitation, or ingestion by biota.^36^ Polymers, such as PE, PP, PS, PET and polylactic acid (PLA), undergo oxidation when exposed to UV light in the environment.^37,38^ Both polyurethane (PU) and PET contain one heteroatom, which undergoes hydrolysis/degradation.^39^ The breaking of ester bonds leads to the development of carboxylic groups, where the acidic nature raises the hydrolysis rate by autocatalytic reaction, as shown in Fig. 3.

The degradation process, such as hydrolysis and photo-oxidation, leads to the making and breaking of bonds. Due to the brittle nature of larger plastic pieces by mechanical stress or friction or abrasion, they were broken into MPs.^40^ The fragmentation pattern of plastics is subject to the environmental circumstances, where the additives present in the plastics could interrupt the physio-chemical properties of the material. For example, Bisphenol A and nonylphenol are antioxidants and UV stabilizers mixed with plastics to protect them from UV light.^41^ Moreover, the fragmentation pattern of plastics was also dependent on the past mechanical stress applied to the plastics during the manufacturing process and utility.^42^ The degradation of plastic waste has been studied, and it was found that the mechanical force could lead to the release of MPs in to the environment.^43–45^ The fragmented plastic debris was found to be reduced in molecular weight, and thereby increased its tendency to be degraded by enzymatic action.^46^ Recent studies indicated that bacterial strains, such as *Bacillus* sp. and *Rhodococcus* sp., had the tendency to degrade PP particles. This observation was tested for 40 days and the degradation efficiency was found to be 6.3%.^47^ However, the fragmentation mechanism of plastic waste has not been studied experimentally, which is still under investigation. The weathering process is expected to start with exterior defects on the plastics, which may lead to breaking. Furthermore, the shear defects, crashes or water disruption could assist in the process.

## 5. Separation of MPs

**Fig. 4:**
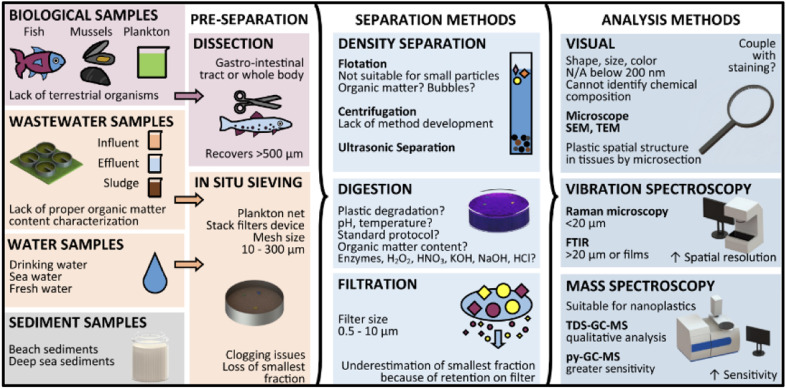
An overview of microplastics separation and analysis methods from simple and complex matrices. Reproduced from ref. 139 with permission from the American Chemical Society, copyright 2019.

Collected ecological samples usually contain impurities, and are not suitable for direct analysis of the MPs. Therefore, it is important to separate and remove the MPs from the sediments. This process can be influenced by the MPs physical and chemical nature, which include the size, structure, density and other sediments. The separation of MPs was carried out by filtration, density separation, biochemical separation, hand picking, sieving and sorting by visual examination. Generally, the separation of MPs from the environmental samples was tough and the shape of the MPs could influence the separation process. Filters with small mesh sizes (0.02–5 μm) were used to separate small MPs and nanoplastics. Similarly, other techniques were applied, such as the elutriation method, where the separation was based on the size, shape and density of the liquid or by a stream of gas which acted in the opposite direction to sedimentation process. The floating process was based on hydrophobic nature and relative buoyancy, which helped to separate the MPs from the environmental sediments^48^. Grbic *et al.*^49^ had attempted to separate the MPs by magnetic method. Herein, the hydrophobic MPs were treated with iron nanoparticles, which was later recovered by a magnet. By this method, they were recovered with a yield of about 92% of PE and PS in the size range from 10–20 μm. MPs with (93%) sizes of 1 mm were recovered, which were made of PET, polyvinyl chloride, PP and PU from the sea water. The efficiency of this separation process was based on the sizes of MPs, extraction process and post density separation of water samples. During this separation process, MPs can be further fragmented. Therefore, the magnetic strength could be specifically tuned based on the polymer. The various steps of common sampling are provided in Fig. 4.

### 5.1 Density separation

**Fig. 5:**
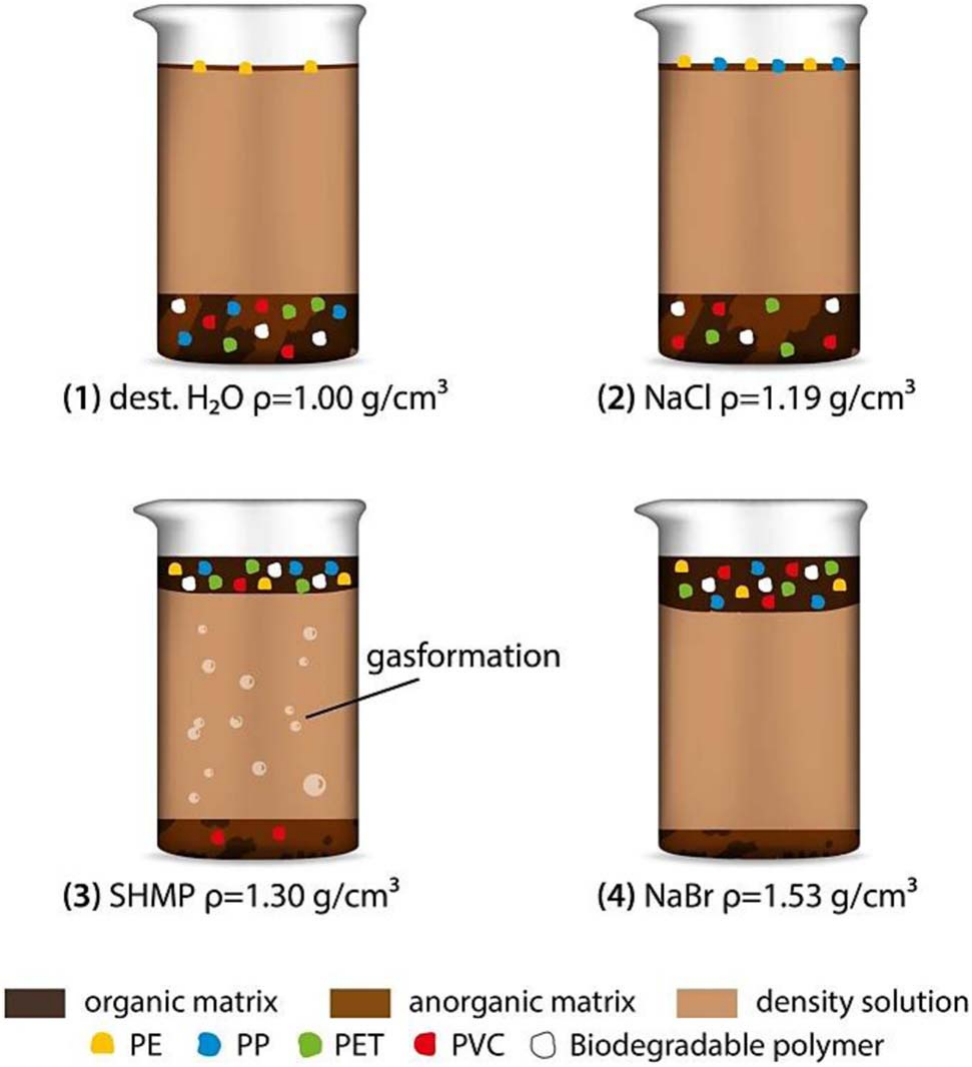
Representation of the density-assisted separation of MPs. Reproduced from ref. 140 with permission from Springer Nature, copyright 2022.

Density separation was a frequently used technique for the segregation and enrichment of MPs from the samples obtained from the environment. MPs refer to small plastic particles that vary in size, often ranging from a few micrometers to a few millimeters.^50^ They were present in several environmental matrices, including aqueous solutions, sedimentary deposits, and terrestrial soil. The use of density separation is advantageous due to the fact that MPs often exhibit distinct densities in comparison to the surrounding materials, hence enabling their separation based on flotation or sedimentation properties.^51^ Density separation was highly efficient, and relied on the distinct densities of MPs. This method ensures the precise isolation of MPs for the accurate identification, as given in Fig. 5. Comparatively, the filtration method offers a simple and cost-effective approach, and is mainly suited for larger MPs, making it practical for initial analysis. Flotation capitalizes on buoyancy, yielding high recovery rates as MPs float while denser materials sink. Flotation was used to separate MPs from denser residues. The densities of commonly used polymers are given in Table 2. Materials with less density would start to float and can be separated easily. By using the liquids (brine solution) with standard density, it was possible to separate the particles which floated at different surface depending on their density.^52^ There are various advantages of using brine solution to separate MPs, such as a quick separation process, repeatable, one step process, less cost and high recoveries of various MPs if their sizes were less than 1 mm. The recovery of MPs from the environmental samples was tested in tap water using different brine solutions made of sodium chloride (NaCl), sodium bromide (NaBr), sodium iodide (NaI) and zinc bromide (ZnBr) (Table 3). As a result, the highest recovery rate of MPs was found when NaI and ZnBr solutions were used with high density polymers in sediments after a single treatment. The sizes of the MPs were found to have some influence on the recovery rate, which had to be considered while choosing the right brine solution for the separation of MPs.

**Table 2:** Common types of polymers and their source and density

S. No.	Classification of polymers	Available forms in the environment^134^	Density (g cm^−3^)^135^
1	PE	Grocery bags, packing sponges	0.926–0.940
2	HDPE	Milk carton, food jars, household cleaners' bottle	0.94 to 0.97
3	LDPE	Container lid, toys, squeeze bottle	0.915–0.925
4	PP	Plastic container, underground storage tanks	0.855–0.946
5	PS	Plastic fork, coffee cup	0.96–1.04
6	PA (nylon)	Thread, raincoat, seat belt	1.13–1.15
7	PET	Soft drinks bottle, water bottle	1.38

**Table 3:** The density of different brine solutions used for the separation of MPs.^135^

S. No.	Solution	Density (g cm^−3^)
1	Water	1.0032
2	Sodium chloride	1.2000
3	Sodium bromide	1.3700
4	Sodium iodide	1.5660
5	Zinc bromide	1.7100

Sediment samples were separated and analysed by using NaCl, which is a cheap and eco-friendly brine solution with the density of 1.2 g cm^−3^. However, it is restricted to polymers with lower density. The elemental composition of each polymer was different, which was analysed by C : H : N analysis to find out the chemical source of the MPs. This separation method narrows down the polymer identification process compared to chemical methods. In this method, the separation was carried out by shaking the sediments in brine solution. This allowed the heavy particles to settle down, while the MPs continue to remain suspended in the brine solution. Later, the MPs present in the upper solution were collected for further analysis. The digestion method was the first and foremost pre-treatment process carried out for the biological samples in order to remove the biological entities from the environmental contaminants. Samples were generally pre-treated by acid digestion, alkaline digestion, or enzyme digestion. In several studies, 35% H_2_O_2_ was used for the digestion of samples, and the results showed that most of the bio-organic components were digested completely.^53^

W. Perren *et al.*,^54^ demonstrated the removal of polymer micro beads from simulated domestic waste water using the electro coagulation method. In this case, they used aluminium electrodes and studied the effect of pH, conductivity and current density. The electrochemical reactor employed was bipolar in a parallel configuration route. Firstly, the polymer microbeads underwent both charge neutralization and flocculation processes. Secondly, due to the combination of the two processes, this electrocoagulation method was cheap with better operation efficiency. This experiment was conducted at pH 7.5 with NaCl (2 g L^−1^) solution. These methods had some advantages compared with density-based separation methods. In the density-based separation, the sizes of MPs could affect the effective separation and identification, and may exclude the tiniest nanoplastics. The other methods (such as filtration and density separation) may require a significant amount of time and efforts, which could have a negative effect on efficiency of the separation when dealing with a large volume of samples.^55^ In addition, the introduction of other impurities or the loss of MPs may occur during the separation process, so it is important to have a controlled environment to ensure the reliability of the investigation.

## 6. Identification of MPs

If the sample collected from the water bodies or soil sediments contains high MPs, direct weighing method can be adopted after removing the impurities. However, if the sample quantity was much less, it is not possible to identify and measure them directly. In this situation, Raman spectroscopy or FT-IR can be used as one of the tools to identify the MPs. These techniques were applied for the confirmation of MPs and to avoid the confusion of the MPs with other organic matters or additional materials present in the sample. Using Raman spectroscopy, MPs with sizes up to 2 mm may be detected. With time-gated Raman spectroscopy, sizes of up to <125 μm and ≥5% mass percentage can be identified.^56^ Micro-Raman spectroscopy also had been employed for the identification of MPs within the size range of 100 μm. Furthermore, particles with a diameter smaller than 10 μm can be effectively detected by micro-Raman spectroscopy and FT-IR spectroscopy techniques.^57^ In FT-IR, the attenuated total reflection (ATR) mode gave the most stable surface spectral data. ATR was mostly used to detect MPs with a particle size of ≥300 μm. The FT-IR spectrum can be recorded within a minute with great accuracy, and was not affected by the fluorescence interference. The infrared light penetrated where the transmission could provide a high-resolution spectrum. The analysis for opaque or thicker samples can be carried out by reflection mode. On the other hand, thermal extraction desorption gas chromatography-mass spectrometry (TED-GC-MS) and pyrolysis-gas chromatography coupled with mass spectrometry (PY-GC-MS) had been used to identify the MPs and the other additives present with it. TED-GC-MS was found to be appropriate for the identification of MPs in environmental samples.^58^ However, extensive sample preparation steps and highly skilled technicians are required to operate it, and these instruments are also highly expensive. Zhang *et al.*,^59^ developed a method for the identification of MPs using the stereomicroscope. In this method, low density MPs were obtained from soil and heated. The number and size of MPs were determined by a developed model. They have proposed that the flotation method was effective for the extraction of MPs from soil with the recovery of 90%.

Some other methods to identify the MPs apart from the expensive techniques include the following: (1) the MPs can be handpicked if the size was higher than one millimeter. (2) If the size is less than one millimeter, it has to be identified by filtration and treatment with Fenton's reagent at a temperature of 40 °C and the pH value of 3, where the organic matters can be washed off from the samples.^60^

### 6.1 Visual identification of MPs

In more than 70% of the reported studies, visual characterization of MPs was the first step in the screening process of the MPs in the environmental samples. This practice involves the physical characteristics of MPs associated with their morphology, colour, *etc.*^61^ MPs (if the sizes are greater than ∼500 μm) can be viewed through the naked eye or light microscope. Recently, some new developments had been reported on the visual detection and testing of MPs, which was carried out with a hot needle. It measured the melting point of the polymer. However, for monitoring MPs or for creating awareness among the general population, the visual detection method was considered. It was also a cheaper and simpler method to confirm the existence of MPs.^62^ There is a need for an upgraded and semi-automated approach for the visual identification of the MPs from the samples obtained. One such approach was reported by using dyes, where the extracted plastics were stained with dyes and quantification of MPs was carried out.^63^ For example, a fluorescent dye (Nile red) was bound with neutral lipids and the synthetic polymers. The stained samples were irradiated with blue light, and MPs were identified and quantified by using fluorescence microscope. Before staining MPs on to the environmental samples, the samples were separated by density-assisted separation method using a brine solution with brisk shaking, and settling overnight to eliminate the proteins and organic matters that may obstruct the fluorescent dyes. The fluorescence of the MPs studied with various colours. Green emission light had three advantages with respect to red and orange. Synthetic polymers had exhibited more fluorescent colours, and organic particles were not fluorescent after digestion under green emission. Therefore, the output signal was less visible. The blue fluorescence gave a strong fluorescent index for man-made polymers. A drawback of this method was that a few plastics/fibres could not be stained by the dye. Therefore, they exhibited very weak signals in the spectrum. The Nile red provided a good recovery rate, which was found to be greater than 95% for MPs with the size of less than 500 μm.^64^ Generally, the samples taken from the sea contain a lot of organic matter (*e.g.*, algae, shells, wood) that are visible to the naked eye. Thus, while staining with dyes, there were some more particles that were found to be inadequately or not stained. Therefore, false positive identification of MPs can be ruled out by comparing it with the visual identification method. Shim *et al.*^65^ reported that the additional staining of organic material could bring a false positive approach while examining MPs. To avoid such misinterpretation, purification steps are required to eliminate the natural organic materials from the samples before the analysis.

**Fig. 6:**
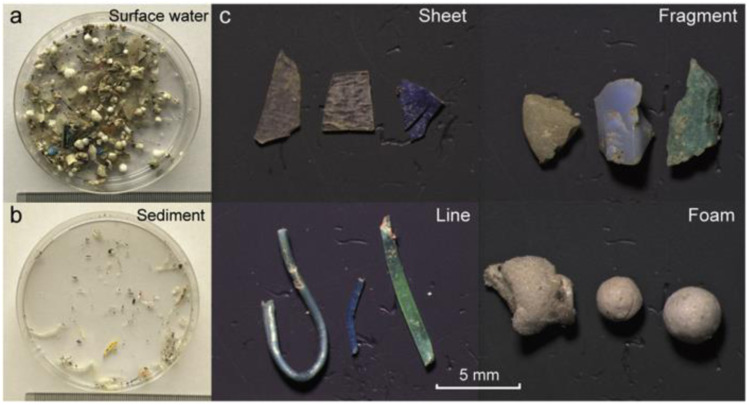
(a) MP samples collected from water; (b) sedimented samples, and (c) separated MPs from the environmental samples in the form of sheets, fragments, lines, and foam. Reproduced from ref. 141 with permission from the American Chemical Society, copyright 2017.

K. Zhang *et al.*^66^ were able to differentiate the MPs by their shapes such as sheet, fragment, line and foam (Fig. 6). These MPs were identified by using Raman spectroscopy. Scanning electron microscopy (SEM) combined with energy dispersive X-ray spectroscopy (SEM-EDS) were used to characterize the morphology of nanomaterials and their elemental compositions. Since most of the MPs were non-conductive, for SEM-EDS analysis, the samples must be properly prepared by the complete drying of samples on a suitable substrate. Subsequently, Au or Pt sputtering will be carried out, which may lead to artifacts and alter the morphological analysis of MPs. Furthermore, due to the usage of a high energy electron beam, a few plastic particles such as polyvinyl acetate (PVA) (melting point of 35–38 °C) and polyvinyl chloride (PVC) (melting point of 115–120 °C) could be softened or charred during the SEM analysis.^67^

### 6.2 Raman spectroscopy

Raman spectroscopy^68^ was used to identify MPs in various environmental samples with high accuracy by applying the frequency shift of inelastic scattered light obtained from the sample based on the Raman effect. As a result, the vibrational modes of the polymers were obtained, which identified the chemical structure of MPs. Meanwhile, all of the synthetic polymers have their own characteristic Raman spectra, so this technique can be used to classify the MPs within a short span of time by comparing the reference spectrum. In biological tissues, the polymers could be identified at the subcellular level by Raman spectroscopy coupled with confocal laser scanning microscopy.^69^ Zada *et al.*^70^ attempted to identify 88 MPs among 12 000 particles per kg taken as dry weight, which was present in the Rhine estuary sediments. Analysis was carried out in less than five hours. This fast analysis was achieved by using stimulated Raman scattering based on the coherent interaction of dual laser beams, along with the vibrational levels in the molecules of the samples obtained. Wolff *et al.*^71^ also identified the MPs (in the form of particles and fibres) by Raman micro-spectroscopy after the chemical and physical purification steps. Firstly, the samples were treated with hydrogen peroxide and sodium hypochlorite to remove the organic matters. Zinc chloride was used for the density separation of MPs.^72^ The diameters of MPs were from 30 μm to 100 μm, and the sizes of MPs fibres were in the range of 100 μm to 1000 μm in length. Most of the MP fibres were made of PET and the MPs (particles), which were generated from PP, PE, PS and PET. Kniggendorf *et al.*^73^ identified the MPs in tap water using Raman spectroscopy with 532 nm laser excitation wavelength, where the particle sizes were found to be 0.1 mm. The identified MPs originated either from PA, PE, PMMA, PS or PP in the form of beads and fragments. Schymanski *et al.*^74^ found MPs in the packaged drinking water and beverage cartons. The sizes of the MPs ranged from 1–500 μm, and identification was carried out by micro-Raman spectroscopy with an excitation wavelength of 532 nm. In this case, gold-coated polycarbonate filters were used for the separation and the sample volume taken was 100 mL per sample. Authors could identify and confirm the presences of the PET, PE, PP and PA. In addition, cellulose was found in the sample, which may have originated from the packaging material.

During the analysis of the MPs, there are a few drawbacks associated with Raman spectroscopy. For instance, the Raman spectrum obtained for the samples with a fluorescent nature may not be interpretable. The baseline variation may occur due to the laser used, which was induced by the fluorescence effect.^75^ Therefore, it was recommended to purify the environmental samples in order to reduce the fluorescence effect on the spectrum before recording the Raman spectra of the samples.^76^

### 6.3 FT-IR spectroscopy

**Fig. 7:**
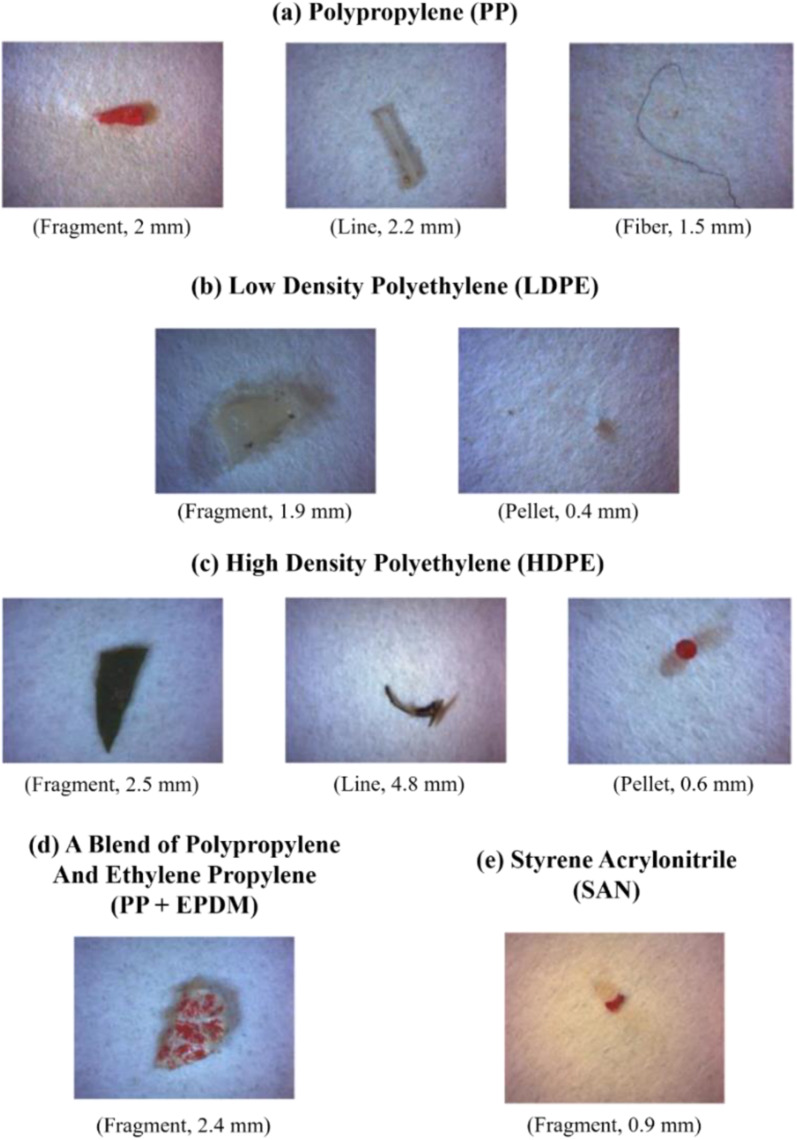
Photographic images of MPs in different shapes: (a) PP, (b) LDPE, (c) HDPE, (d) combination of PP and ethylene propylene, and (e) SAN. These MPs were extracted from the surface water and identified by ATR/FT-IR. Reproduced from ref. 142 with permission from Elsevier, copyright 2017.

**Fig. 8:**
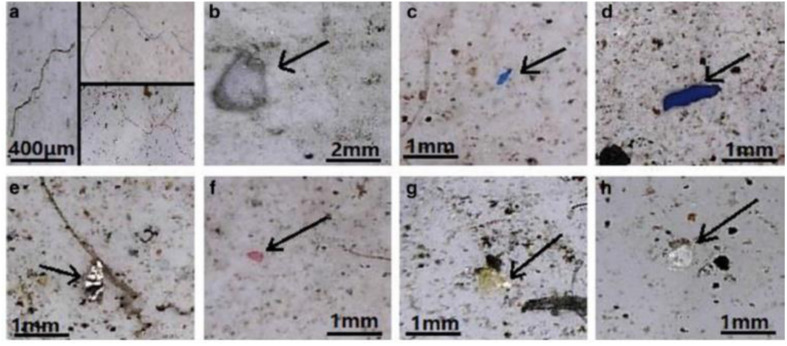
Optical microscopy images of MPs. (a) MP fibres, (b) PS foam, (c and d) PP fragments and (e–h) PE films. Reproduced from ref. 143 with permission from Springer Nature, copyright 2017.

**Fig. 9:**
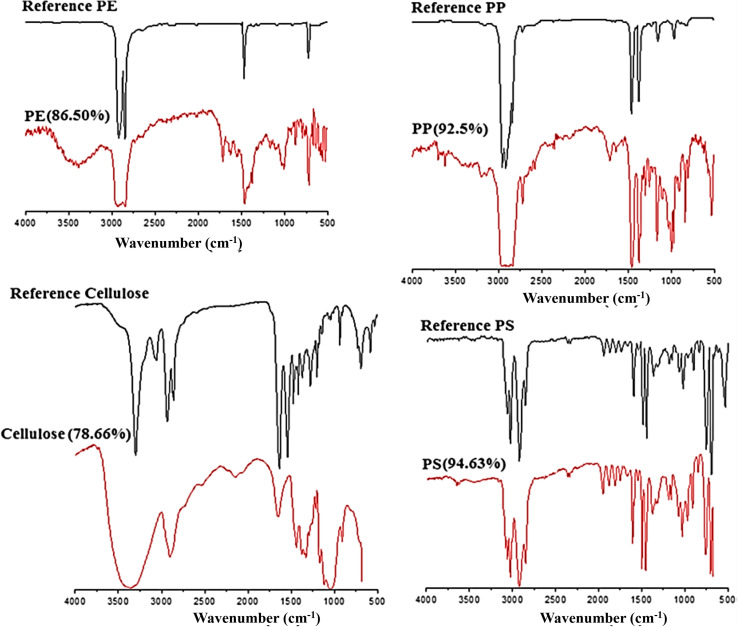
FT-IR spectra of the identified MPs (PE, PP, cellulose, and PS), which were matched with the known standard spectra. Reproduced from ref. 143 with permission from Springer Nature, copyright 2017.

FT-IR spectroscopy could provide accurate identification of MPs because each polymer has its unique characteristic infrared spectrum. FT-IR was useful for the qualitative analysis of MPs when the sizes ranged from 10 μm. MPs can be detected using two different modes by FT-IR measurements, such as transmittance and reflectance. Tagg *et al.*^77^ detected the MPs by using Focal plane array (FPA) based reflectance FT-IR (FPA/FT-IR), which identified various synthetic polymers in the size ranging from 150–250 μm in the sewage water plant samples. In this case, samples first underwent pre-treatment with 30% H_2_O_2_ to eradicate biogenic matters. From this study, MPs were identified as PE, PP, PS, PVC and nylon 6. The main advantage of FPA/FT-IR spectroscopy was that it could give fast results in a short span of time (in less than nine hours of time), and a circular filter with 47 mm of diameter was used for filtration. Tsang *et al.*^78^ used attenuated total reflectance (ATR/FT-IR) spectroscopy to identify the functional groups of MPs. The sediment and water samples were collected and analysed, which indicated the presence of PP, LDPE, HDPE, a combination of PP/ethylene, propylene and styrene acrylonitrile (SAN). These MPs were present in the sample as (shapes) line, fragments, pellet, and fibres (Fig. 7). Another study was carried out by L. Cai *et al.*^79^ in the environmental samples collected after washing with ultra-pure water and drying at 50 °C for 48 h. They confirmed and identified the presence of MPs in various shapes, include pellets, fibre, and rods. In addition, micro-FT-IR showed the presence of PE, PP, PS, and cellulose in the selected samples (Fig. 8 and 9). Mason *et al.*^80^ performed the detection and identification of MPs in the packaged water. The MPs were identified after Nile red tagging using FT-IR spectroscopy. The density of MPs was found to be 325 particles per L, and most of the MPs were present as PS, PE, and PA.

### 6.4 Hyperspectral imaging

Hyperspectral imaging (HSI) is a novel, simple and quick method to identify the MPs in the environmental sediments. The HSl imaging method demonstrated its exceptional capability in distinguishing MPs from natural materials owing to their distinct spectral fingerprints. This method offered comprehensive data pertaining to the dimensions and categorization of MPs, which was of utmost importance in the evaluation of their ecological ramifications. The use of this non-destructive methodology ensures the preservation of sample integrity, while exhibiting a remarkable level of sensitivity, enabling the detection of MPs even at low concentrations. In addition, it has significant geographical mapping functionalities, hence assisting in the detection of hotspots.^81^ HSI was found to be an effective method to distinguish the polymers from organic particles in the sea water.^82^ However, HSI contains many redundant and highly inter-related spectral details, which resulted in the Hughes phenomenon for sorting the spectrum. The HSI provided the support vector machine (SVM) algorithm^83^ model output, which was said to have good recovery rate and accuracy for the detection of various MPs with different particle sizes. The assessment of MPs was based on infrared HSI analysis in the wavelength range of 900–1700 nm. Zhu *et al.*^84^ identified MPs using the HSI method, where eleven categories of MPs were identified by their characteristic spectra observed in the different wavelength range of 1150–1250 nm, 1350–1450 nm, and 1600–1700 nm. In this study, the gold-coated polycarbonate filter was used for the loading of MPs. The hyperspectral images were usually recorded in three modes, *i.e.*, point scanning, line scanning and plane scanning. Moreover, the HSI method was used to estimate particle sizes and shapes of polymers (such as PP, PS and PE) in both the soil and aquatic environment. Some of the drawbacks of the HSI method was associated with its operation difficulty and data processing. HSI acquisition and maintenance might be costlier. HSI data analysis is a computationally demanding process that calls for certain tools and comprehension. It is not suitable for field or on-site or use in isolated areas. Insufficient depth penetration makes it more difficult to find deeply buried MPs. Hyperspectral system operation can be complicated, requiring knowledge of technology and measurement. Its spatial resolution may not be appropriate for ongoing observation. A model or a well-known material is required to standardize the pixel information, which can be used to examine the unknown samples *via* a model transfer procedure.^85^

### 6.5 NMR spectroscopy

Peez *et al.*^86^ suggested a method to detect and quantify the MPs using ^1^H NMR spectroscopy. They had identified the LDPE granules, PET fibres and PS beads. The accuracy was attained using the calibration curve method. The limit of detection (LOD) was found to be from 19–21 μg mL^−1^, and the limit of quantification (LOQ) was 74 to 85 g mL^−1^. This method could be used to identify and quantify the MPs within this concentration range. Compared to other detection methods, such as Raman spectroscopy, FT-IR and GC-MS, NMR is a size-independent detection method.^87^ There were no maximum or minimum particle sizes required for the detection of MPs since all the MPs were dissolved in a suitable solvent, and then analysis was carried out. However, the environmental samples must be pre-treated to remove the biological matters and inorganic deposits by digestion process. N. Peez *et al.*^87^ identified the LDPE granules, PET fibres, and PS beads, and also quantified them by NMR spectroscopy. Before analysis, the samples were cleaned using sulfuric acid and hydrogen peroxide *via* chemical digestion. After that, the samples were dried at 60 °C. The solvent used for NMR was CDCl_3_/trifluoroacetic acid (4 : 1) at room temperature. This method also provided a high recovery rate for each analysis.

### 6.6 Pyrolysis-gas chromatography-mass spectrometry (PY-GC-MS)

PY-GC-MS is a technique that is used for the identification of MPs and reveals its polymer type by examining the sample after the thermal degradation process. This is one of the destructive techniques. Moreover, 5–200 μg of sample can be examined in one measurement. Using this method, polymers can be identified along with the organic plastic additives present in the sample.^88^ The MPs obtained from wastewater-treated samples were sorted out under a microscope. These separated samples were further analysed by PY-GC-MS.^89^ The environmental samples contained wires, pellets and fragments of the polymers, such as PE, PS and ethylene/propylene rubber (EPDM). Dehaut *et al.*^90^ stated that the PY-GC-MS technique was very useful for the identification of the polymer types, and may not differentiate the polymers based on their density. By using the PY-GC-MS technique, we can find the mass of the polymers. So, the pre-selection of MPs by visual optical sorting technique was still required. The sample must be grinded well in a cryomill to achieve good results. This analysis was carried out at the time of pyrolysis under an oxygen-free environment after the combustion. The obtained product was analysed by GC-MS. As an alternative of this technique, a new process was developed which was known as thermos-extraction and desorption couples with gas chromatography mass spectrometry (TED-GC-MS).^91^ This technique was the combination of thermogravimetric analysis and thermal desorption gas chromatography, where we can perform rapid quantification of the common polymers, such as PE, PS, PP, and PA6 in the environmental samples. It was possible to obtain promising composition results without the pre-assortment of the samples. Similarly, Fischer and Scholz-Böttcher^92^ proposed a method based on Curie point PY-GC-MS and thermos-chemolysis method, where identification of eight common polymers present in the samples were demonstrated. In this case, no sample pre-sorting or pre-treatment was required, and the recovery was also done by spiking the known polymers into fish samples, which gave accurate results. Nuelle *et al.*^93^ identified MPs in marine sediments using PY-GC-MS, and reported the presence of various polymers, such as PP, PVC and PET. Before analysis, the air-induced overflow/flotation technique based on two-step extraction with NaCl was used for pre-extraction to reduce the original sediment sample mass. In addition, NaI was used for the subsequent flotation of MPs, which was found to be effective for identification of common polymers in marine sediments.

## 7. MPs impact on aquatic organisms

The accumulation of MPs on finfish could be predicted by one of the ingestion methods that was based on feeding patterns and further grouping them.^94^ In this connection, Daniel *et al.* detected the presence of MPs in non-edible parts, such as viscera, gills, *etc.*, and in edible tissues, such as skin and muscles of pelagic fish. The total accumulation ratio in edible tissue was more than 41%^95^ since there are two types of feeding mechanisms in pelagic fish, among which filter feeding fishes engulf huge volumes of plankton entangled with MPs, which was then trapped in their gills and further transferred to the esophagus. This kind of passive feeding leads to accidental intake of PE, which was found in higher amount in some demersal and pelagic finfish, specifically in their gills and viscera.^96,97^ In another study, a lesser number of MPs was reported in their skin tissues and muscles when compared to gills and viscera accumulation.^98,99^ In addition to that, Saturno *et al.*^100^ demonstrated the presence of MPs in commercially available finfish that were collected infield. Pacific herring (*Clupea pallasii*) that was collected from Salish sea contained microfibers. The area where the sample fish was collected was a highly urbanised one,^101^ whereas the finfish that was collected along the remote coastal area showed the absence of microfibers. In another study, researchers from Mexico had examined stress in carnivorous, omnivorous and an herbivorous finfish due to MPs, which indicated an abundant content of MPs in the omnivorous fish compared with the other two groups.^102^

Fibre-like materials were the most recorded microplastic form in wild caught finfish, and that were further ingested in other higher marine organisms.^103,104^ Finfishes, such as salmon, *Carassius carassius*, rockfish and pike, accumulated the MPs in their tissues. Also, they were observed to relocate MPs from hepatic tissues to gastrointestinal tract.^105^ MPs could cause several problems such as low energy reserves, oxidative changes in the predatory behaviour, hepatic stress, and decreased lipid metabolism and various physiological effects.^106–108^ In the experimental condition, *Dicentrarchus labrax* (European sea bass) was exposed to polluted and untreated PVC MPs pellets for a time period of 30–90 days, which resulted in pathological changes in the epithelium of its intestine, which could further create health issues.^109^ If there was a short-term exposure of the same species, a minimum negative effect was observed.^110^ It was also studied that the variation might be due to different experimental setups, such as the types of MPs selected and their exposure periods and concentrations.^111^

**Fig. 10:**
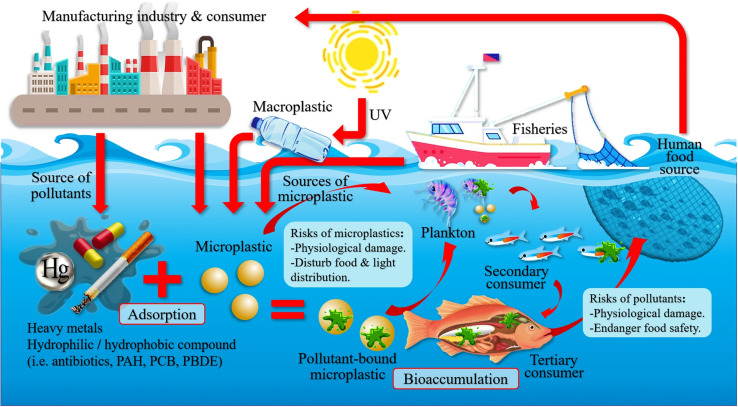
The global impact of microplastics on the marine ecosystem and organisms. Reproduced from ref. 144 with permission from Springer Nature, copyright 2021.

In the case of the shellfish organism, *Cerithidea obtusa* (mangrove snail) collected from Jambi, Sumatera and Pangkal Babu were reported to have 167 MPs.^112^ Piarulli *et al.*^113^ had reported on the 117 particles of MPs in the digestive tract of crab *Carcinus aestuarii* located in Adriatic Sea. This study was conducted to estimate different kinds of MPs accumulated in edible crab. Similarly, Ningrum & Patria found four types of MPs fibres, which were majorly presented in shellfish, and have been documented to have a positive correlation between the body weight of crab and snail with the abundance of MPs.^114^ Since the fibre has a relatively lesser density, it remains floating on the water surface for a longer period of time, and the granules sink due to its higher density. To date, the well represented commercial group of fisheries related to microplastic research was carried out with bivalves due to its feeding modes, sessile nature, cultural and ecological importance. The blue mussel of *Mytilus edulis* is one of the bivalve species considered for microplastic studies in experimental condition, since they are a sediment-dwelling organism.^115^ Furthermore, shell fish such as lobster, mussels, clams and oysters were collected from estuarine and coastal ecosystems in North America and studied for the MPs presence in their tissues.^116^ There were a few studies that demonstrated that microplastic beads, pellets, and fibres were taken in by scallops, mussels, oysters and clams.^117^ This study also revealed that the accumulation of MPs could vary with respect to their organ and the global impact of MPs on the marine ecosystem and organisms (Fig. 10). For example, the American oyster which also had the highest commercial value was reported for the accumulation of nanoplastic beads in its hepatopancreas.^118^ This report said that in that part of North America, the bivalves were exposed to different materials, types and sizes of MPs with the mixed biological endpoints and physiological effects.^119^ There are many shapes of MPs such as microfibers, and microbeads that could cause an elevated rate of respiration, condensed fecundity, neurotoxicity, DNA damage, and changes in feeding habits in all of the individual species.^120^ However, Kolandhasamy *et al.*^121^ studied the MPs with 100 μm sizes that were incorporated into different organs of blue mussels. They also had indicated that MPs accumulation was not only present in organs associated with the digestive system, but also in the mantle, foot, *etc.*, which was due to the direct contact of MPs by mussels through unknown mechanisms. This study observed that the blue mussels had the potential to eliminate MPs by the process of depuration of its gut in filtered seawater that resulted in 60% of egestion over 9 h from the total MPs ingested.^120^

## 8. Limitations of identification and quantification of MPs

Lack of standardization for MPs analysis:

• Absence of standardized methods for the identification and quantification of MPs.

• Variation in sample collection, depth, volume, density, and water content reporting.^122^

Analytical method challenges:

• Aberrations, contaminants, and misconceptions due to augmented analytical methods.

• The need for careful monitoring during the identification and quantification of MPs.

• The importance of using blank samples as controls^123^

Analytical method validation:

• The requirement for parameters like precision, accuracy, specificity, selectivity, and limit of detection for method validation.

• The need to follow European Commission Decision 2002/657/EC for method authentication.

• Observations of lack of reproducibility in some cases.^124^

Statistical relevance:

• The importance of statistical relevance for the effective evaluation of MPs in the environment.

• The impact of the differences in surface, texture, and dimension between chemical and polymeric quantification on the analytical process.^125^

### 8.1 Challenges in analysing MPs

Difficult sampling process:

• The challenge of collecting representative samples of MPs from the environment.

• The need to develop dynamic and thorough sampling approaches for accurate evaluation.^126^

Reliability in laboratory *vs.* Real site conditions:

• Inconsistencies in reported values for MPs concentrations in the environment.

• Lack of reliability between laboratory conditions and real-site concentrations.

• Environmental samples often differ in size, colour, and shape from uniform laboratory samples.^127^

Spectra variation in polymers:

• Differences in polymer spectra in contact with environmental samples.

• Surface changes and the generation of new functional groups.

• The importance of recording MPs contamination with diverse polymer spectra under certain conditions of biological degradation.^128^

Method development and quality:

• Ongoing changes and improvements in MPs identification and quantification methods.

• The need to select high-quality methods based on the research question and study objectives.^126^

## 9. Future prospect

The future of MPs research holds promising prospects in several key areas. Researchers will work towards the standardization and enhancement of methods for microplastic analysis. This involves establishing universally recognized protocols for sample collection, preparation, and analysis, promoting consistency and enabling cross-study comparisons. Advanced analytical techniques will also evolve, enhancing accuracy and efficiency in the identification and quantification of MPs. These improved methods, coupled with innovative sampling approaches, will enable the comprehensive capture of MPs in diverse environmental matrices, while optimizing cost-effectiveness. Quality control and reproducibility will be paramount, with the development of rigorous quality assurance and quality control procedures to bolster result reliability. As MPs continue to pose threats to ecosystems, understanding their transformations and behaviours in real-world environments, including UV exposure and biological degradation, will be a top research priority.

Interdisciplinary collaboration will provide a holistic perspective on microplastic impacts. Researchers will engage with policymakers and the public to drive changes in plastic waste management practices, emphasizing recyclability and reforming plastic production processes. Long-term monitoring programs will track changes in microplastic contamination over time, offering insights into trends and the effectiveness of mitigation strategies. In this way, the future of microplastic research aims to not only save ecosystems, but also promote a sustainable approach to plastic materials through recycling and reforming practices.

## 10. Conclusions

The proliferation of MPs in water bodies worldwide has become an urgent scientific concern. This escalating contamination necessitates a comprehensive approach, encompassing rigorous research, awareness dissemination, and the implementation of effective protocols. Scientific investigations into MPs have predominantly relied on visual identification methods to distinguish them from non-plastics, a valuable initial step for rapid screening. However, this approach carries the risk of sample count inaccuracies and classification errors, particularly when natural fibres are misinterpreted as MPs. Our article addresses this challenge by exploring innovative analytical methods for precise MPs identification and quantification in environmental samples. However, our scientific journey is far from complete. A deeper understanding of polymer toxicity, kinetics, and the development of standardized protocols for evaluation of MPs in seawater is very critical. It is essential to acknowledge that all analytical methods have their strengths and limitations. A thorough scientific examination is needed to unravel how weathering and biological degradation processes affect the ecological cycle in the presence of MPs. Moving forward, our scientific direction is clear. We must refine our MPs assessment methods and establish a universally accepted, scientifically rigorous approach. By fostering interdisciplinary research, adhering to scientific standards, and raising awareness, we can mitigate the global consequences of MPs and work toward a sustainable solution to this ecological challenge.

## Author contributions

P. M.: conceptualization, methodology, writing, reviewing and editing. P. S. & S.·B.: methodology, writing, reviewing and editing. R. A.: methodology, writing, reviewing and editing. S. A.: writing, reviewing and editing. A. K. S.: conceptualization, funding, supervision, methodology, writing, reviewing and editing.

## Conflicts of interest

The authors declare no competing interests.

## Supplementary Material
